# Evaluation of factors for predicting risk of uveitis recurrence in Behcet's disease patients

**DOI:** 10.1590/1414-431X20209118

**Published:** 2020-05-08

**Authors:** Jianfei Cai, Lin Qi, Yong Chen, Jun Zou, Yan Shen, Dan Luo, Huafang Bao, Jingfen Ye, Haifen Ma, Jian-Long Guan

**Affiliations:** 1 Department of Rheumatology and Immunology, Huadong Hospital Affiliated to Fudan University, Shanghai, China; 2 Department of Radiology, Huadong Hospital Affiliated to Fudan University, Shanghai, China; 3 Shanghai Key Laboratory of Clinical Geriatric Medicine, Huadong Hospital Affiliated to Fudan University, Shanghai, China

**Keywords:** Anterior uveitis recurrence, Behcet’s disease, Oral ulcers, Triglycerides, Low-density lipoprotein, Serum amyloid A

## Abstract

This study aimed to investigate the predictive factors for uveitis recurrence (UR) risk in Behcet's disease (BD) patients. BD patients (n=164) with a history of uveitis were recruited, and demographic data, clinical features, and laboratory tests were recorded. Uveitis was defined as anterior uveitis, intermediate uveitis, posterior uveitis, panuveitis referring to the “International Uveitis Study Group recommendations for the evaluation of intraocular inflammatory disease”. In total, there were 70 UR patients and 94 non-UR patients. Compared to non-UR patients, UR patients appeared to be older and presented with increased uveitis occurrence rate and times within 3 months, oral ulcers occurrence rate, as well as higher concentrations of triglycerides (TG), total cholesterol (TC), low-density lipoprotein (LDL), and serum amyloid A (SAA). Multivariate logistic model disclosed that uveitis occurrence times within 3 months, oral ulcers, TG, LDL, and SAA independently predicted higher risk of UR. Furthermore, receiver operating characteristic curve analysis showed that the combination of uveitis occurrence times within 3 months, oral ulcers, TG, LDL, and SAA exhibited a high predictive value for UR risk with an area under the curve of 0.983 (95%CI: 0.969−0.998). In conclusion, uveitis occurrence times within 3 months, oral ulcers, TG, LDL, and SAA might be potential predictive factors for UR risk in BD patients, which can help in prevention and management of the disease.

## Introduction

Behcet’s disease (BD) is a severe systemic debilitating vasculitis featuring recurrent oral aphthous ulcers, genital ulcers, uveitis, as well as skin lesions, which usually involves the musculoskeletal system, eyes, nervous system, vascular beds, gastrointestinal tract, and cardio-pulmonary system (1). Ocular involvement, one of the most common factors of disability caused by BD, has been reported to affect nearly 70% of BD patients (1). As the most common manifestation of ocular involvement, uveitis is a chronic recurrent disease with characteristics of explosive attacks of severe inflammation that can lead to damage of the intraocular structures (2). Although great advances in uveitis treatment have been achieved, the recurrence of uveitis is still high and uveitis recurrence (UR) might bring about great anguish physically and mentally in BD patients; long-term treatment also provides huge financial burden (1). Thus, investigation of predictive factors for UR risk is essential for effective treatment and prognostic improvement in BD patients.

Due to the rareness of BD, few studies have explored the predictive factors for organ involvement risk in BD patients, and only a few have proposed that the percentage of Treg cells, presence of brainstem atrophy in initial magnetic resonance imaging, and increased scintigraphic uptake occurring in symptomatic acral joints by bone scintigraphy act as useful predictive factors for ocular, neuro, and joint involvement in BD patients, respectively (3–5). However, for the predictive factors for UR risk in BD patients, little is known. In order to address this aspect, this present study enrolled 164 BD patients with a history of uveitis, with the purpose to investigate the predictive factors for UR risk in BD patients.

## Material and Methods

### Patients

Between January 2016 and August 2018, 164 BD patients with a history of uveitis treated at Huadong Hospital Affiliated to Fudan University were recruited for this study. The inclusion criteria were as follows: i) previously diagnosed as BD according to the International Criteria for Behcet’s Disease (ICBD, 2006) or the revised ICBD Criteria (2010) (6); ii) had a history of uveitis complicated with BD; iii) age above 18 years, and iv) had no serious infection. Patients were excluded from the study if they were presenting with intermediate uveitis, posterior uveitis, or panuveitis, or complicated with metabolic disease, neoplasms, hypertension, or diabetes mellitus. The approval of this study was obtained from the Ethics Committee of Huadong Hospital Affiliated to Fudan University, and informed consents were signed by all patients prior to recruitment.

### Data collection

At enrollment, demographic data, clinical features, and laboratory tests of patients were recorded, including age, gender, uveitis occurrence times within 3 months, oral ulcers, white blood cells (WBC), alanine aminotransferase (ALT), aspartate aminotransferase (AST), triglycerides (TG), total cholesterol (TC), high-density lipoprotein (HDL), low-density lipoprotein (LDL), blood urea nitrogen (BUN), creatinine (Cr), glycated hemoglobin A1c (HbA1c), prothrombin time (PT), activated partial thrombin time (APTT), potassium (K), sodium (Na), erythrocyte sedimentation rate (ESR), C-reactive protein (CRP), and serum amyloid A (SAA).

### Assessment of UR

Recurrence of uveitis was identified by an experienced physician based on the anatomic criteria of uveitis including iritis, iridocyclitis, anterior cyclitis, pars planitis, posterior cyclitis, hyalitis, basal retinochoroiditis, and focal, multifocal, or diffuse choroiditis, chorioretinitis, retinochoroiditis, or neurouveitis, and panuveitis, which was in accordance with “International Uveitis Study Group recommendations for the evaluation of intraocular inflammatory disease” (7). Among 164 recruited patients, there were 70 patients presenting with recurrence of uveitis, which were defined as UR patients, and the other 94 patients without recurrence of uveitis were defined as non-UR patients.

### Treatment

According to the clinical status, all patients received appropriate treatment, which was as follows: 1) ciclosporin+thalidomide+infliximab (ciclosporin 50 mg bid, thalidomide 25-50 mg qd, and infliximab 3 mg/kg) and 2) prednisone+ciclosporin+thalidomide+infliximab (prednisone 5.0-7.5 mg qd, ciclosporin 50 mg bid, thalidomide 25-50 mg qd, and infliximab 3 mg/kg).

### Statistical analysis

Data analysis was performed by SPSS 21.0 (IBM, USA), and figures were plotted by GraphPad Prism 6.01 (GraphPad Software Inc., USA). Difference of characteristics between two groups was compared by the *t*-test or chi-squared test. Relationships of variables with the recurrence of uveitis were determined by the univariate logistic regression analysis; the variables with independent influence on the recurrence of uveitis were screened by the forward stepwise (conditional) multivariate logistic regression analysis, and the risk prediction model of the recurrence of uveitis was constructed based on the independent influencing variables. Then the predictive performance of the risk prediction model was further assessed by the receiver operating characteristic (ROC) curve and the derived area under the curve (AUC). P<0.05 indicated a significant difference.

## Results

### BD patients’ characteristics

The mean age of BD patients was 31.62±6.24 years, and there were 113 males and 51 females (Table 1). The number of BD patients with uveitis within 3 months was 95 (57.9%) and the mean uveitis occurrence time within 3 months was 1.19±1.28. Totally, there were 70 UR patients and 94 non-UR patients. Compared to non-UR patients, UR patients were significantly older, and presented with higher uveitis occurrence within 3 months, more uveitis occurrence times within 3 months, increased oral ulcers occurrence, higher TG concentration, higher TC concentration, higher LDL concentration, and higher SAA concentration. No significant differences in the other characteristics were found between UR patients and non-UR patients. In addition, there were 15 (9.1%) patients (including 9 (12.9%) UR patients and 6 (6.4%) non-UR patients) receiving ciclosporin+thalidomide+infliximab and 149 (90.9%) patients (including 61 (87.1%) UR patients and 88 (93.6%) non-UR patients) receiving prednisone+ciclosporin+thalidomide+infliximab. There was no difference in treatment choice between UR patients and non-UR patients (P=0.155). The detailed information is shown in Table 1.

### Factors affecting UR by univariate logistic regression analysis

Univariate logistic regression analysis revealed that age, uveitis occurrence times within 3 months, oral ulcers, TG, TC, LDL, and SAA were correlated with increased risk of UR in BD patients (Table 2). In addition, there was no correlation of treatment choice with UR risk in BD patients.

### Factors affecting UR by multivariate logistic regression analysis

Further forward stepwise (conditional) multivariate logistic regression analysis disclosed that uveitis occurrence times within 3 months, oral ulcers, TG, LDL, and SAA could independently predict higher risk of UR in BD patients (Table 3).

### Predictive value of factors for UR risk by ROC curves

**Figure 1:**
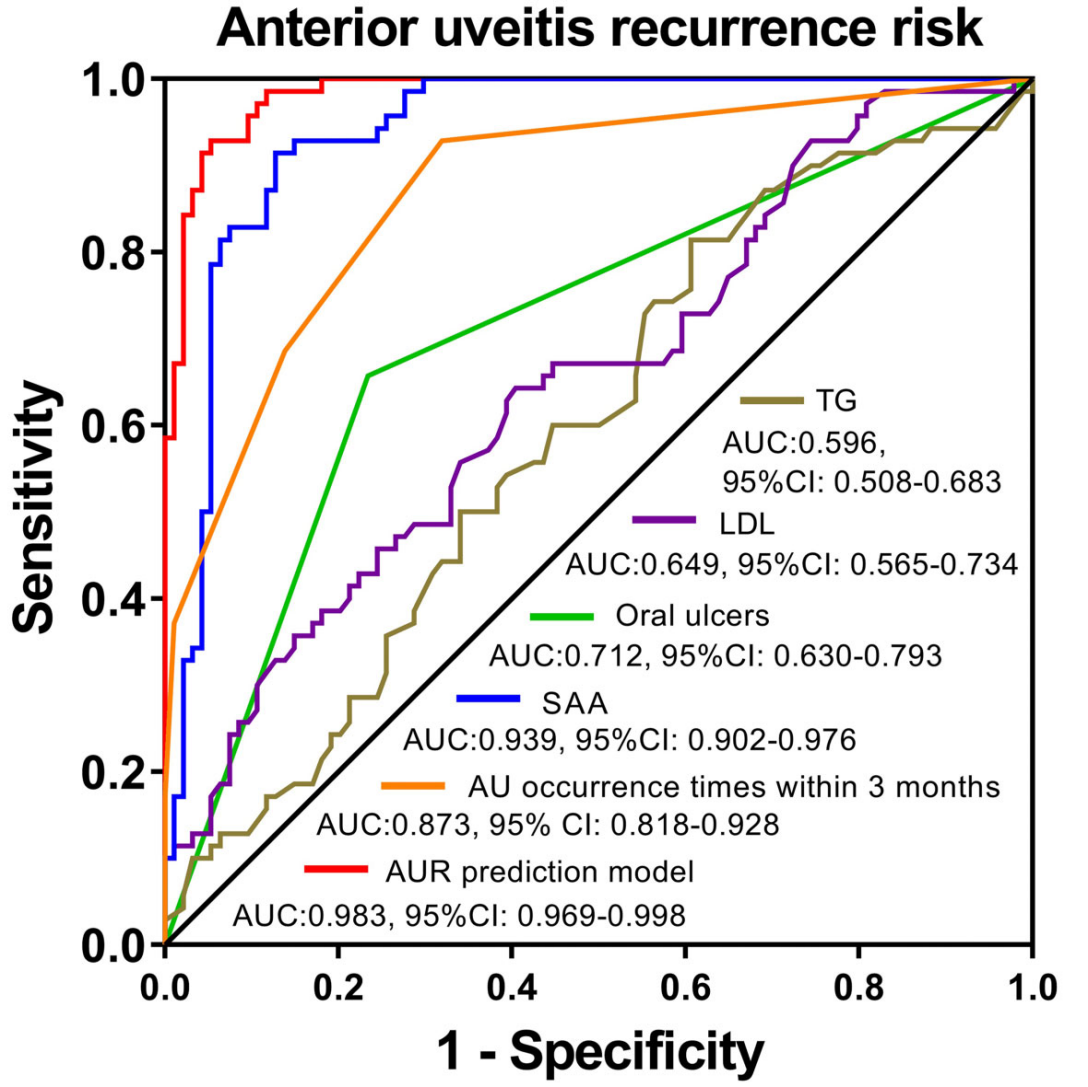
Predictive value of factors for UR risk in Behcet’s disease patients. The ROC curves and the derived AUC were used for assessment of the predictive performance of factors. ROC: receiver operating characteristic; UR: uveitis recurrence; AUC: area under the curve; TG: triglycerides; LDL: low-density lipoprotein; SAA: serum amyloid A. UR prediction model where -2ln(LR)=52.680:

Subsequently, we used ROC curves to evaluate the predictive value of the above-mentioned factors from forward stepwise (conditional) multivariate logistic regression analysis for UR risk in BD patients, which showed that TG, LDL, oral ulcers, uveitis occurrence times within 3 months, as well as SAA could distinguish UR patients from non-UR patients, among which SAA appeared to have the best predictive value for UR risk (Figure 1). In order to further evaluate the total predictive value of these 5 factors for UR risk, we constructed the UR prediction model. The ROC curve disclosed that the UR prediction model presented a great predictive value for UR risk in BD patients with AUC of 0.983 (95%CI: 0.969-0.998) (Figure 1).

$$P=\frac{e[1.415 (uveitis occurrence times within 3months)+2.273 (Oral ulcers)+3.111 (TG)+1.839 (LDL)+0.342 (SAA)-24.188]}{1+e[1.415 (uveitis occurrence times within 3months)+2.273 (Oral ulcers)+3.111 (TG)+1.839 (LDL)+0.342 (SAA)-24.188]}$$

**Table 1:** Characteristics of Behcet’s disease patients.

Items	Total patients (n=164)	UR patients (n=70)	Non-UR patients (n=94)	P value
Age (years)	31.62±6.24	33.11±5.97	30.51±6.23	0.008
Gender				0.443
Male	113 (68.9)	63 (55.7)	50 (44.2)	
Female	51 (31.1)	27 (52.9)	24 (47.1)	
Uveitis occurrence within 3 months	95 (57.9)	27 (52.9)	30 (31.9)	<0.001
Uveitis occurrence times within 3 months	1.19±1.28	2.16±1.19	0.47±0.76	<0.001
Oral ulcers	68 (41.5)	46 (65.7)	22 (23.4)	<0.001
WBC (x10^9^/L)	6.65±1.47	6.90±1.48	6.46±1.45	0.060
ALT (U/L)	28.96±2.96	28.73±2.98	29.13±2.95	0.395
AST (U/L)	22.70±5.57	21.76±5.38	23.40±5.63	0.061
TG (mM)	1.50±0.34	1.56±0.40	1.45±0.28	0.028
TC (mM)	5.16±0.81	5.40±0.81	4.97±0.76	<0.001
HDL (mM)	1.39±0.35	1.36±0.35	1.40±0.35	0.455
LDL (mM)	3.11±0.70	3.34±0.73	2.94±0.62	<0.001
BUN (mM)	5.42±0.83	5.55±0.84	5.33±0.82	0.097
Cr (μmol/L)	68.00±13.96	67.37±14.56	68.47±13.56	0.617
HbA1c (%)	5.49±0.29	5.50±0.29	5.49±0.30	0.755
PT (s)	11.98±0.83	12.03±0.88	11.95±0.80	0.535
APTT (s)	33.68±1.14	33.61±1.16	33.73±1.13	0.507
K (mM)	4.03±0.35	4.04±0.41	4.03±0.31	0.904
Na (mM)	143.43±1.58	143.44±1.61	143.43±1.56	0.945
ESR (mm/h)	13.60±6.25	14.39±5.53	13.02±6.70	0.156
CRP (mg/L)	4.28±1.50	4.25±1.51	4.29±1.50	0.857
SAA (mg/L)	28.76±9.50	36.73±4.91	22.82±7.55	<0.001
Treatment				0.155
Ciclosporin+thalidomide+infliximab	15 (9.1)	9 (12.9)	6 (6.4)	
Prednisone+ciclosporin+thalidomide+infliximab	149 (90.9)	61 (87.1)	88 (93.6)	

Data are reported as mean±SD or number with percentage in parentheses. Comparisons between groups were determined by *t*-test or chi-squared test. UR: uveitis recurrence; WBC: white blood cells; ALT: alanine aminotransferase; AST, aspartate aminotransferase; TG: triglycerides; TC: total cholesterol; HDL: high-density lipoprotein; LDL: low-density lipoprotein; BUN, blood urea nitrogen; Cr, creatinine; HbA1c: glycated hemoglobin A1c; PT: prothrombin time; APTT: activated partial thrombin time; K: potassium; Na: sodium; ESR: erythrocyte sedimentation rate; CRP: C-reactive protein; SAA: serum amyloid A.

**Table 2:** Univariate logistic regression analysis of factors affecting anterior uveitis recurrence.

Parameters	Univariate logistic regression			
	P value	OR	95%CI	
			Lower	Higher
Age	0.009	1.072	1.018	1.129
Gender (male)	0.444	0.785	0.422	1.459
Uveitis occurrence times within 3 months	<0.001	4.839	3.062	7.646
Oral ulcers	<0.001	6.273	3.157	12.465
WBC	0.062	1.229	0.990	1.525
ALT	0.393	0.955	0.860	1.061
AST	0.062	0.947	0.895	1.003
TG	0.036	3.140	1.075	9.174
TC	0.001	2.063	1.347	3.161
HDL	0.453	0.709	0.288	1.743
LDL	<0.001	2.445	1.485	4.028
BUN	0.097	1.377	0.943	2.011
Cr	0.614	0.994	0.972	1.017
HbA1c	0.753	1.184	0.412	3.400
PT	0.533	1.126	0.775	1.636
APTT	0.504	0.911	0.694	1.197
K	0.903	1.056	0.439	2.537
Na	0.944	1.007	0.827	1.226
ESR	0.167	1.036	0.985	1.090
CRP	0.856	0.981	0.798	1.207
SAA	<0.001	1.408	1.261	1.573
Treatment				
Ciclosporin+thalidomide+infliximab	Reference	−	−	−
Prednisone+ciclosporin+thalidomide+infliximab	0.163	0.462	0.156	01.365

OR: odds ratio; CI: confidence interval; WBC: white blood cells; ALT: alanine aminotransferase; AST: aspartate aminotransferase; TG: triglycerides; TC: total cholesterol; HDL: high-density lipoprotein; LDL: low-density lipoprotein; BUN, blood urea nitrogen; Cr: creatinine; HbA1c: glycated hemoglobin A1c; PT: prothrombin time; APTT: activated partial thrombin time; K: potassium; Na: sodium; ESR: erythrocyte sedimentation rate; CRP: C-reactive protein; SAA: serum amyloid A.

**Table 3:** Forward stepwise (conditional) multivariate logistic regression analysis of factors affecting anterior uveitis recurrence.

Parameters	Multivariate logistic regression			
	P value	OR	95%CI	
			Lower	Higher
Uveitis occurrence times within 3 months	<0.001	4.116	1.909	8.872
Oral ulcers	0.007	9.705	1.864	50.518
TG	0.026	22.434	1.451	346.798
LDL	0.010	6.289	1.549	25.539
SAA	<0.001	1.408	1.215	1.633

OR: odds ratio; CI: confidence interval; TG: triglycerides; LDL: low-density lipoprotein; SAA: serum amyloid A.

## Discussion

BD is an obliterative and necrotizing vasculitis, impacting both the arteries and veins in all organ systems, which is particularly prevalent in the ‘Silk Route’ populations and it appears to be strongly related to the geographical area of BD patients’ residence (1). Due to the rareness of BD, only a few studies have been performed to investigate the predictive factors for organ involvement in BD patients. For instance, an interesting study discloses that increased scintigraphic uptake occurring in symptomatic acral joints by bone scintigraphy presents with predictive value for joint involvement in BD patients (5). Meanwhile, the presence of brainstem atrophy in the initial magnetic resonance imaging has been displayed to have potential in predicting a progressive course for neuro involvement in BD patients (4). Also, serum TC, TG, and LDL is increased, but amylin is decreased in BD patients compared to controls, and amylin level is negatively correlated with parameters of metabolic syndrome, active manifestation, as well as corticosteroid dose, indicating that amylin could be a factor for the development of metabolic syndrome in BD patients (8). Meanwhile, serum preptin and amylin is low, and both of them serve as potential factors for development of metabolic syndrome in BD patients (9).

Ocular involvement is the most serious manifestation of BD and it is usually featured by recurrent non-granulomatous uveitis with necrotizing obliterative retinal vasculitis, which may lead to secondary glaucoma, cataract formation, macular edema, or even disability (10,11 [Bibr B12] [Bibr B13] [Bibr B14] [Bibr B15]). As the most common manifestation of ocular involvement, uveitis is a chronic recurrent disease featured with explosive attacks of severe inflammation that causes great damage in the intraocular structures (1,10,11). Although improvements have been achieved in uveitis treatment, there is a high recurrence of uveitis, and UR could result in a devastating reduction in quality of life and productivity in BD patients (1). Only one previous study recruited 19 BD patients with ocular complications, and disclosed that the percentage of Treg cells in CD4+ T cells from these patients were decreased before ocular attack compared with those after ocular attack, and decreased percentage of Treg cells may be a predictive marker of ocular attack in BD patients (3). Considering the small sample size in that previous study, the statistical power might be poor.

We herein enrolled 164 BD patients with a history of uveitis, which was the largest sample size for predicting UR risk until now (our center was the largest center with the highest numbers of BD patients in China), and we explored the predictive factors for UR risk in these BD patients. The findings revealed that uveitis occurrence times within 3 months, oral ulcers, TG, LDL, and SAA were independent predictive factors for higher risk of UR in BD patients. The possible explanations were as follows: 1) uveitis occurrence times within 3 months not only directly reflect the recurrence of uveitis in BD patients, but also indirectly imply that the short-term control might be unstable. Therefore, these patients were more prone to UR; 2) for oral ulcers, it was one of the most common manifestation of BD and it indirectly reflected the severe course of BD, thus, the risk of UR might be increased with the worse course of BD; 3) high concentration of TG and LDL meant a high risk of hyperlipidemia, increased blood viscosity, or even embolus that cause vessel stenosis, which might accelerate inflammatory factors’ accumulation and increased risk of UR in BD patients. In addition, treatments for reduced inflammation (such as high dose glucocorticoid treatment) could induce the secretion of VLDL by the liver and the production of hydroxychloroquine to decrease TG and LDL levels, suggesting that decreased inflammation was related to decreased TG and LDL (12-15); and 4) SAA, a precursor substance of tissue amyloid protein A belonging to acute phase protein, could be elevated in response to tissue damage and inflammation that affect cell adhesion, migration, proliferation, and aggregation. Thus, the high value of SAA reflected severe inflammation, thereby increasing the risk of UR in BD patients.

In addition, we discovered that TG, LDL, oral ulcers, as well as uveitis occurrence times within 3 months could distinguish UR patients from non-UR patients. More importantly, SAA could distinguish UR patients from non-UR patients with AUC of 0.939 (95%CI: 0.902−0.976), which might be due to the strong correlation of SAA with inflammation. Moreover, the ROC curve showed that our UR prediction model had a great predictive value for UR risk with AUC of 0.983 (95%CI: 0.969−0.998). These findings provided new evidence for predicting the risk of UR in BD patients with a history of uveitis, which might help to improve their prognosis.

Although several interesting findings were discovered in this study, there were still limitations. Selection of BD patients with a history of uveitis from a single hospital and a retrospective design were the main limitations of this study, which might cause selection bias and confounding bias. Therefore, a further prospective multicenter study with more patients is necessary.

In summary, uveitis occurrence times within 3 months, oral ulcers, TG, LDL, and SAA could serve as independent predictive factors for higher risk of UR, and the combination of these five factors presents a good predictive value for UR risk in BD patients.
